# Perspectives on Intra- and Intercellular Trafficking of Hedgehog for Tissue Patterning

**DOI:** 10.3390/jdb4040034

**Published:** 2016-12-02

**Authors:** Eléanor Simon, Adrián Aguirre-Tamaral, Gustavo Aguilar, Isabel Guerrero

**Affiliations:** Centro de Biología Molecular “Severo Ochoa”, Universidad Autónoma de Madrid, CSIC-UAM, Nicolás Cabrera 1, Cantoblanco, 28049 Madrid, Spain; esimon@cbm.csic.es (E.S.); adrian.aguirre@cbm.csic.es (A.A.-T.); gustavo.aguilar@estudiante.uam.es (G.A.)

**Keywords:** Hedgehog, cytonemes, gradient formation, pattern formation, modeling

## Abstract

Intercellular communication is a fundamental process for correct tissue development. The mechanism of this process involves, among other things, the production and secretion of signaling molecules by specialized cell types and the capability of these signals to reach the target cells in order to trigger specific responses. Hedgehog (Hh) is one of the best-studied signaling pathways because of its importance during morphogenesis in many organisms. The Hh protein acts as a morphogen, activating its targets at a distance in a concentration-dependent manner. Post-translational modifications of Hh lead to a molecule covalently bond to two lipid moieties. These lipid modifications confer Hh high affinity to lipidic membranes, and intense studies have been carried out to explain its release into the extracellular matrix. This work reviews Hh molecule maturation, the intracellular recycling needed for its secretion and the proposed carriers to explain Hh transportation to the receiving cells. Special focus is placed on the role of specialized filopodia, also named cytonemes, in morphogen transport and gradient formation.

## 1. Introduction

The evolutionary conserved Hedgehog (Hh) proteins are secreted ligands, which trigger the Hh signaling pathway upon binding to their receptor complex present at the cell surface. Hh was brought to light in a screen for genes involved in *Drosophila* embryonic patterning more than 35 years ago [1]. Hh signaling controls different cellular processes: cell survival, division, differentiation, cell migration or axonal pathfinding (reviewed in [2]). It is secreted from a localized source, the producing cells, to reach the receiving cells to activate its targets in a concentration-dependent manner. 

The interest of such a mechanism is that a single molecule is sufficient to genetically specify different domains in an undifferentiated field. Hh homologues have been identified in other organisms. In vertebrates, three homologues, Sonic hh (Shh), Indian hh (Ihh) and Desert hh (Dhh), have been described. Among other things, the development of the limb and neural tube in vertebrates or of the wings in flies constitute suitable models to tackle the establishment of the Hh morphogen gradient.

Hh undergoes post-translational modifications by covalent addition of two lipid moieties (cholesterol and palmitic acid), which constrain the molecule by firmly tethering it to the lipid bilayer of the producing cells, ruling out the possibility of free diffusion. For this reason, the understanding of the mechanism underlying Hh transport from producing to receiving cells is one of the current challenges. Several hypotheses have been proposed to explain how Hh is able to move across the extracellular matrix to reach its target cells, such as micelle-like structure formation [3], association with lipoproteins [4] or with exosomes [5,6]. A different conceptual view for Hh delivery is the cytoneme-mediated transport, which permits the Hh signaling molecule to attain its complex receptor while being associated to thin actin-based filopodia emitted from the producing cells. Thus far, cytoneme-mediated Hh transport has been observed in the wing imaginal disc [6,7,8], in the abdomen [8] and in the germarium of the ovary [9] of *Drosophila*, and in the limb bud [10], in the retina and the neural tube neuroepithelia [11] of vertebrates. Cytonemes are labile and dynamic structures up to 150 µm long [10]. Importantly, alteration of cytonemes formation affects Hh gradient length, pointing out a functional role for these specialized signaling filopodia in the proper delivery of Hh [8]. In addition, vesicles containing Hh moving along cytonemes are visualized, suggesting a probable release as exosomes when cytonemes interact with the receiving cells [6].

In *Drosophila*, after Hh reaches the receiving cell, it is recognized by the receptor complex formed by Patched (Ptc) (the first described Hh receptor) [12], and the adhesion molecules Interference hedgehog (Ihog) and Brother of ihog (Boi) [13]. In vertebrates, the receptor complex is formed by Ptc1, CAM-related/downregulated by oncogenes (Cdo), Brother of CDO (Boc) and Growth arrest-specific 1 (Gas1), the latter lacking an ortholog in *Drosophila* [14]. In addition, the transducer of the signal Smoothened (Smo) and the downstream transcription factor Cubitus interruptus (Ci) in *Drosophila*, the Gli family in vertebrates, constitute the core components of the pathway and the base for understanding Hh signaling and its regulation (reviewed in [2]).

This review revisits the mechanism of Hh synthesis, maturation and secretion in the light of recent discoveries. We also describe the mechanisms involved in Hh transport that allow the morphogen to reach the receiving cells, with special interest on the contribution of cytonemes as a scaffold to control the transport of vesicles loaded with Hh. A statement of the evolution of mathematical models used to explain morphogen distribution is also provided, showing a biophysical approach to Hh signaling. Finally, contradictory results within the field and unsolved relevant questions about cytonemes will be discussed.

## 2. Hedgehog Synthesis, Lipid Modification and Shedding

After the amino-terminal signal peptide is hydrolyzed, Hh enters into the secretory pathway. Then, Hh precursor undergoes an autoproteolytic cleavage in the endoplasmic reticulum [15,16], which releases two fragments: the Hh C-terminal region, containing the catalytic activity required for cleavage that is instantly sent to the proteasome for degradation [17], and the Hh N-terminal region, bearing the signaling activity [18]. This Hh N-terminal region is sterified by cholesterol on the C-terminal in the same cleavage reaction [17] and a palmitic acid is added on the conserved N-terminal cysteine residue [19] catalyzed by the acyltransferase Skinny hedgehog [20]. The addition of both lipid residues is required for optimal activation of the pathway [21,22,23]. These post-translational modification events give rise to a highly liphophilic molecule, which coerces its tethering to the cell membranes [24,25], in particular, to the lipid rafts’ microdomains at the plasma membrane [26,27], regions rich in cholesterol and sphingolipids. 

Interestingly, lipid modifications on Hh are absolutely required for the formation of the Hh gradient (reviewed in [28]), and it is still an open question of how Hh overcomes its strong association with the cell membrane of producing cells to move to the receiving cells. Genetic studies in both flies and vertebrates have identified the gene *dispatched* (*disp*) as essential for Hh release [29,30,31,32,33]. *disp* encodes for a twelve-pass transmembrane protein, which shares homology with the resistance-nodulation-cell division superfamily (RND) of bacterial efflux pumps, which power transport of cholesterol-derived molecules across the membrane bilayer utilizing a proton gradient as a source of energy [34]. Moreover, Disp has the particularity to contain a sterol-sensing domain (SSD), a cellular lipid sensor domain and a typical motif of lipid metabolism regulatory enzymes [29,31]. Although Hh binds physically to Disp [33,35] via its cholesterol moiety [36], it remains unknown whether or not this interaction involves a specific contact between the SSD domain of Disp and the cholesterol-modified Hh. Alternatively, the SSD domain may be important for Disp localization within the lipid rafts [29] and/or for its vesicular trafficking [33]. In mutant cells for Disp, lipid-modified Hh is not released from the producing cells, and only a juxtacrine signaling is activated [29,31]. However, artificial lipid-free Hh is released in Disp mutant cells, indicating that Disp mediates specifically lipid-modified Hh secretion [29,33].

Vertebrate cells also require the secreted glycoprotein Scube2 (signal peptide, cubulin domain, epidermal-growth-factor-like protein 2) for the release of cholesterol-modified Shh into the culture medium, but the release of non-cholesterol-modified Shh is Scube2-independent as in the case of *disp* mutant cells [32,36,37,38,39,40]. Its mechanism of action is controversial. Although it is clear that Hh binds Disp before being yielded to Scube2, it has been suggested that Scube2-mediated Hh release is stimulated by the cholesterol moiety [36], or by the palmitate residue [40]. Scube2 is proposed to capture Shh from a Disp-bound form [36], supporting the idea that cholesterol is a tether that must be severed to liberate Hh [36,40,41,42,43]. A process mediated by a disintegrin and metalloprotease (ADAM) protein has been suggested for the proteolytic removal of N- and C-terminal lipidated peptides [41,42]. In fact, Scube2 enhances N- and C-terminal Shh shedding from the surface of Shh producing cells [44]. However, this shedding of Hh only occurs after the interaction of its N-terminal part with its receptor Ptc, as palmitate moiety seems to be needed for Hh–Ptc interaction [43], suggesting that Hh is still attached to the presenting membrane when interacting with Ptc in the receiving cells. The vertebrate mechanism cannot be easily translated to *Drosophila.* Flies lack a Scube2 ortholog [32,37,38], and a proteolytic removal of N- and C-terminal lipidated moieties by shedding has not been described. In this context, it would be interesting to test the role of the secreted factor Shifted (Shf), especially since this protein is known to contribute to Hh release only when Hh is lipid-modified [7,45,46,47,48,49]. 

## 3. Apicobasal Polarity of Hh Gradient and Trafficking Regulators

New models for Hh transport and release for tissue patterning have emerged based on the observation of Hh distribution in the monolayer of the wing imaginal disc epithelium. Hh immunostaining reveals that two different Hh pools (apical and basolateral) are transported to the receiving cells. However, the relative contribution of each pool to long- and short-range signaling remains controversial. Two antagonist models have been proposed. 

**Figure 1:**
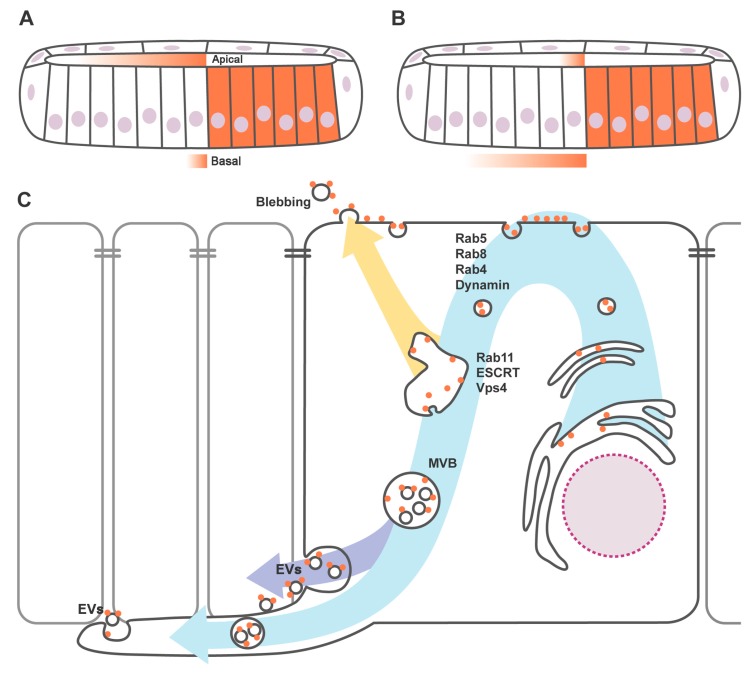
Schematic representation of Hedgehog (Hh) secretory trafficking and apicobasal polarity of the Hh gradient. (**A**,**B**) Scheme of a transversal section of a wing imaginal disc to show the two proposed models of Hh gradient formation. One model proposes that apically secreted Hh contributes to short-range signaling while basolaterally secreted Hh forms the long-range gradient formation [7,33] (**A**); alternative model suggests that apical Hh fraction activates long-range signaling while Hh basal fraction contributes to short-range signaling [50,54] (**B**); (**C**) Hh secretory trafficking. Hh is secreted after synthesis, processing and lipid modifications in endoplasmic reticulum. The Hh apical pool is then internalized in a process in which Rab5, Dynamin and Rab8 are implicated [33,54,63]. After this endocytic event, Hh follows the Multivesicular body (MVB) pathway to be secreted basolaterally (**violet** arrow) and probably travels associated to cytonemes (**light blue** arrow). These exovesicles (EV) most probably travel inside cytonemes [6,8]. Alternatively, other reports propose that the internalized Hh pool from the apical membrane is re-secreted apically (**yellow** arrow) involving, among others, endosomal sorting complexes required for transport (ESCRT) machinery and Vacuolar protein sorting-associated protein 4 (Vps4), and that Hh EV are formed via blebbing of the apical plasma membrane without implications of cytonemes in Hh gradient formation [5,50].

One proposes [50] that the apical Hh pool is responsible for the long-range targets’ activation, while the basolateral pool activates short-range targets (Figure 1A). Among other observations, this is mainly based on the apical but not basolateral sequestration of Hh far from its source by a Ptc mutant form defective for internalization [51,52].

Others [33] suggest that the apical pool is implicated in juxtacrine signaling, contributing to the activation of the short-range targets, while the basolateral pool is responsible for long-range Hh gradient formation (Figure 1B). This conclusion was initially based on the accumulation of ligand and receptor in the receiving cells of the wing disc epithelium when the endocytosis is blocked with a thermosensitive mutant form of Dynamin; Hh and Ptc colocalize several cell diameters away from the source at the basolateral plasma membrane. In contrast, only one cell diameter of Hh–Ptc colocalization is found at the apical region of the receiving cells under the same mutant condition. 

The formation of an apical or basal Hh gradient could depend on how producing cells present Hh to receiving cells. The recycling model proposed by Callejo et al. [33] suggests that, in the producing cells, Hh is secreted apically, followed by autocrine internalization, apical to basolateral intracellular transport and ligand presentation (Figure 1C). Accordingly, interfering with endocytosis in Hh producing cells results in the accumulation of Hh in the apical domain [33,50,53] and the depletion of Hh in the basolateral membrane, with a subsequent shortening of the long-range signaling [33,53]. Interestingly, not only Dynamin, but also Rab5, Rab4 and Rab8 are implicated in the first step of recycling and the disruption of their function causes Hh subapical accumulation and a reduction in the range of target induction [33,54].

After apical internalization, Hh travels back to the plasma membrane in a process in which Disp might be implicated [33,55]. It has been shown that Disp is localized basolaterally in *Drosophila* [33] and in vertebrate polarized epithelia [35], suggesting that Hh is released from the basolateral membrane. Glypicans have also been demonstrated to undergo transcytosis in the wing disc [7,56,57]. Mutant cells for the glypican Dally-like (Dlp) phenocopy Disp mutant phenotype. Both proteins physically interact and colocalize, suggesting a functional interaction between Disp and glypicans for Hh intracellular trafficking [33]. Analogous apicobasal trafficking has been proposed for Wingless (Wg) [57,58] and for the Epidermal growth factor (EGF) ligand Spitz [59], indicating a conserved mechanism for the basolateral release of lipid-modified signaling molecules. 

The role of the glypican Dally during Hh secretion is also controversial. According to the recycling model for Hh basolateral release, Dally has been proposed to be a key factor in the retention of Hh at the apical surface of producing cells, permitting its subsequent internalization and then its basolateral presentation [7]. In contrast, others have proposed that Dally upon cleavage of its Glycophosphatidylinositol (GPI) anchor could mediate Hh apical release [60,61]. This is all based on the ectopic expression of a non-physiological secreted form of Dally (without its GPI anchor) [60,61], and also based on the consideration of Notum as a Glypican lipase [61] needed to mediated Hh apical release. However, Notum does not cleavage Dally GPI anchor and is not needed for Hh release [62].

## 4. Hedgehog Loaded in Exovesicles

Hh does not disperse as monomeric units but in a higher packaging organization [3,64,65,66]. To explain Hh transport of several soluble carriers, whose lipoproteic composition, size and subcellular origin differ, larger-scale clusters [67,68], lipoprotein particles [4,23] or argosomes [69] have been proposed. In addition, vehicle independent transport mechanisms have been reported, in which Hh could be solubilized via its own oligomerization [64,65]. It has also been reported that Hh gradient depends on its secretion via exovesicles (EV) [5,6,63].

Several pathways for EV formation have been described (reviewed in [70]). In particular for Hh EV secretion, two mechanisms have been suggested: microvesicle blebbing/shedding [5] and multivesicular body (MVB)-mediated secretion [6,63,71] (Figure 1C). Given that both pathways require endosomal sorting complexes required for transport (ESCRT) machinery [72], the discrimination between both types of EV constitutes an important challenge. It is not known whether endocytosis is required for blebbing-mediated EV formation. However, the MVB formation is believed to start with an endocytic event. In fact, it has been demonstrated in vitro and in vivo that the loading of Hh in EV requires a previous internalization process [63,71]. Moreover, Hh endosomal accumulation has been reported as a result of disruption of the ESCRT machinery, both in cell culture and in vivo [6,63,71], showing that it is at the MVB level where the ESCRT machinery participates in the loading Hh in EV.

It is also remarkable that interfering with Sphingomyelinase (SMase), a major regulator of ESCRT-independent EV formation [73], causes a reduction of Hh targets in vivo, suggesting that ESCRT-independent and -dependent formation of Hh EV could coexist [6]. Interestingly, the ability of purified Hh EV for target induction in the tissue culture system is incomplete [6], indicating that a more complex scenario is needed to simulate an in vivo system. Thus, EV could be either coexisting with other Hh vehicles or EV delivery requires extra machinery for presentation to the receiving cells.

## 5. Cytoneme-Mediated Hh Transport

Since filopodia-mediated morphogen transport was first proposed [74], several groups have reported this kind of transport for different signaling molecules such as Bone morphogenetic protein (BMP) [75,76], Wnt [77,78], EGF [79], Fibroblast growth factor (FGF) [79], Notch [78,80,81,82,83] and Hh [8,9,10]. These specialized signaling filopodia were named as cytonemes (from the Greek *cyto*: cell, and *nema*: thread). The elusive behavior of these structures (they are often disrupted by chemical fixation and also highly dynamic) has hampered its description. However, the irruption of live imaging techniques [8,10,84] enables deciphering the role of cytonemes in cell–cell signaling during development.

Cytonemes are proposed to drive Hh dispersal both in *Drosophila* [8,9] and in vertebrates [10]. In *Drosophila* wing and abdominal epithelia, Hh basolateral cytonemes extend several cell diameters from producing to receiving cells. Reduction of cytoneme length by temporally knocking down actin polymerization factors such as suppressor of cAMP receptor (SCAR) or Capping protein subunits in the Hh producing cells correlates with shortening of the gradient [8]. Similarly, interfering with the function of the formin Diaphanous (Dia) or Wiskott-Aldrich Syndrome protein (WASP), both key regulators of filopodial actin bundle formation, causes loss of Hh target activation in *Drosophila* germ cells [9]. These results indicate that Hh signaling depends on the proper functioning of these structures.

Studies in vertebrates suggest a conserved role for MyosinX (MyoX) in Shh [10] and Wnt cytoneme transport [77]. In both cases, MyoX is associated to cytoneme tips, but not to Shh or Wnt moving vesicles, indicating a structural function of MyoX in cytoneme formation (as reported for filopodia formation in cell culture [85]). Surprisingly, *Drosophila* does not have a MyoX ortholog, and it might be possible that another myosin supplies its function.

It is clear that cytoneme growth requires an interaction with the extracellular matrix components, in particular with Heparan Sulfate Proteoglycans (HSPGs). Experiments performed in the *Drosophila* epithelium revealed that inhibition of HSPG function in Hh receiving cells, by means of the removal of the exostosin (EXT) members genes *tout velu* (*ttv*) and *brother of ttv* (*botv*), which encode two glycosyltransferases necessary for the HSPG synthesis [86,87,88,89], impedes the basal invasion of cytonemes from Hh producing cells. Cytonemes get stuck at the edge of double *ttv*, *botv* mutant clone [8]. As a result, Hh cannot reach its target cells and the activation of the pathway is restricted in a juxtacrine manner to the first row of *ttv* and *botv* mutant cells touching the anteroposterior compartment border [21,86,88,89]. This result supports the proposal of cytonemes-mediated delivery of Hh and reveals that cytonemes use extracellular matrix components for their stabilization [8].

Proteins involved in Hh presentation, such as Disp, Shf, Ihog or Dlp, are present in cytonemes [7,8,33]. Alteration in some of these components has been demonstrated to modify cytoneme behaviour and Hh gradient. The overexpression of the Hh coreceptors Ihog and Boi in Hh producing cells reduces drastically cytoneme dynamics and Hh gradient [7,8], indicating a dependence between Hh presentation machinery and cytoneme structural components [8]. 

How Hh moves along the filopodia remains controversial. Live imaging of Hh and Ihog positive vesicles labeled with Cluster of Differentiation 63 (CD63) (a marker of exosomes and MVB in mammalian cells [4,90]) shows that these vesicles travel within filopodia towards the receiving cells [6]. Alternatively, it has been proposed that Hh moves in extracellular vesicles on the surface of cytonemes [10]. This kind of “surfing” has been reported for EGF containing particles [91] and for exosome uptake [92].

## 6. Vesicular Trafficking for Hh Reception

Hh reception also plays a role in the extracellular gradient formation just by simple uptake and degradation of the signaling molecule to limit its spreading [52,89,93,94]. Ptc, the main Hh receptor, is a twelve-pass transmembrane protein [12,95] and binds Hh proteins [52,96,97,98]. As Disp, it is evolutionarily related to the RND family of channels and transporters [99], also contains a SSD domain [100,101,102,103] and is localized to lipid rafts [104]. Mutants in the SSD of Ptc affect its vesicular trafficking and its interaction with the transducer of the signal Smo [102,103].

Other Hh coreceptors in *Drosophila* are Ihog and Boi. Both molecules [104,105], together with the glypican Dlp, but not Dally [13,89,106,107,108], are absolutely required for Hh reception. Ptc [52,93,94,109], Ihog and Boi [105,110], Dlp [57] and Dally [61] were found to have a clear involvement in the recruitment and/or internalization of Hh at reception to define a concentration gradient. Significantly, these molecules have similar roles in Hh-producing and Hh-receiving cells, but the outcome is different in each cell population, something that has also been revealed by the functional analysis of DSulfatase-1 (DSulf1) [111]. It has been proposed that DSulf1, by its desulfation catalytic activity, lowers the Hh/HSPG interaction in Hh source and target fields, thereby enhancing the release of Hh from its source and reducing Hh signaling activity in the responding cells.

Thus far, most of the identified molecules required for Hh reception are located in cytonemes emanating from the receiving cells [7,8,33], although Ptc is only visualized in cytonemes when endocytosis is blocked because of its high internalization speed [8]. Supporting a mechanism of cytoneme-mediated Hh signaling [8,10], Hh would be transported bound to membranes and only released by shedding after recognition of the receptor Ptc in the receiving cytonemes [42]. Whether receiving and producing cytonemes contact between each other at the tip or along filopodia, or whether this contact takes place between cytonemes and the cell body, remains unknown. 

The model of cytoneme-mediated Hh transport, release and reception indicates that the initial binding and uptake of Hh occurs at the basolateral part of an epithelium. In contrast, in vertebrates, it has been proposed that the primary cilium, specialized cytoplasmic extension located in the apical part of a polarized cell, is essential for Hh signaling [112,113,114,115]. However, the site of Hh signal transduction and activation processes in this structure may not be the initial site of signal uptake [116].

## 7. Modeling for Morphogen Dispersion

### 7.1. Classical Modeling Approach

**Figure 2:**
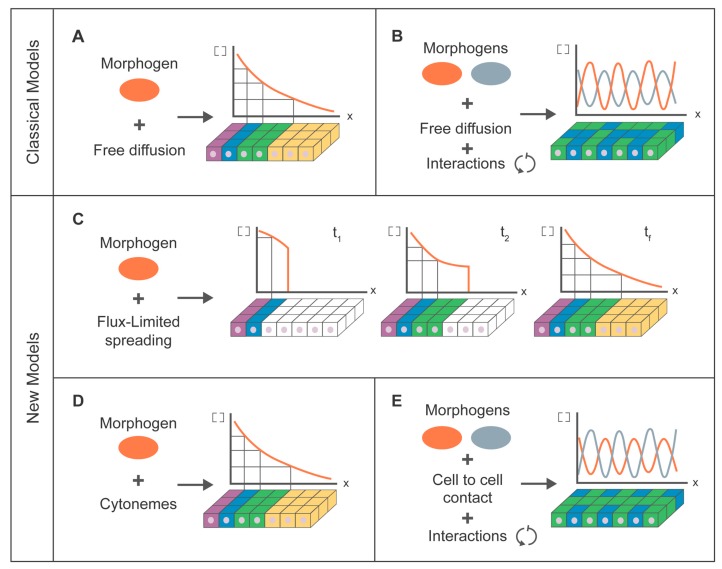
Schematic representation of the main similarities and differences between the mathematical frameworks of molecular signaling. (**A**) Classical view of a signaling gradient through free diffusion equation (positional information theory) [118]; (**B**) Turing model, taking into account the free diffusion of two morphogens and the interactions between them [119]; (**C**) Evolution of the gradient states at different times (t_1_,t_2_) until the final state (t_f_) under the flux-limited spreading model, considering a nonlinear mechanism of transport (speed limitation) due to the properties of biochemical molecules do not allow them to move freely [127]; (**D**) Theoretical approximation to a specific biological mechanism (cytonemes) to explain the experimental gradient shown in C [128]; (**E**) Signaling model through cell-to-cell contact and interaction between morphogens to explain pattern formation [81].

Modeling approaches have been applied in biology for many years, as a result of a collaborative effort between biologists, mathematicians and physicists. These approaches are especially significant to explain pattern formation in biology since the first works of Turing and Wolpert. Gradient formation has been simulated from a biophysical perspective using linear diffusion equations (free diffusion associated with Brownian processes) [117] due to the simple adaptation of the mathematical equations of diffusion to the theory of positional information (French flag model) described by Wolpert in 1969 [118]; when there is a gradient, the distribution of a morphogen in a tissue determines cell fate in a concentration-dependent manner (Figure 2A). However, in general, the development of biological systems does not depend on a unique morphogen but rather the pattern is a result of interactions between two morphogens acting as activator and/or repressor. This biological behaviour was described theoretically in 1952 [119] when Turing demonstrated mathematically the possibility to establish a precise spatial pattern through the interaction of molecules (later named morphogens) (Figure 2B). These reaction–diffusion equations have been applied to different biological systems [120,121], some related to the Hh family of proteins such as feather formation [122], lung branching [123], ruggae in the mammalian palate [124] and long bone development [125]. The mathematical frameworks to explain Wolpert´s gradients or Turing’s tissue patterning are quite similar [121]. Both models explain general concepts of the signaling processes through linear diffusion and both lose consistency if the hypothetic molecules do not diffuse freely due to their biochemical properties, as is the case for Hh.

### 7.2. Cytoneme Modeling Approach

In parallel with the different biological hypothesis proposed for Hh transport, different mathematical models have been developed to support them [126]. In what follows, the focus will be directly on those models concerning cytonemes. One such approach combines biological data and a new mathematical equation, called flux-limited spreading (FLS) equation, in order to explain Hh gradient formation [127]. It takes into account the fact that some molecules cannot freely move by linear diffusion in a biological medium (usually the extracellular matrix). A nonlinear mechanism for morphogen transport (in this case, cytonemes) imposes a restricted velocity of propagation. This velocity has experimentally been measured in two different paradigms (tissue culture cells and wing imaginal disc of *Drosophila*). The FLS equation can make predictions regarding the concentration front of Hh morphogen and the dynamics of response of the gradient during developmental time (Figure 2C). In contrast, classical diffusion equations need an artificial non-biological threshold in the concentration of protein levels to match the theoretical predictions to experimental biological results. This new FLS equation paves the way to model gradient formation through nonlinear mechanisms, but needs improvement to provide a more accurate mathematical framework to model the behaviour of cytonemes.

A modeling combining experimental data on cytonemes with a simulation of the formation of the gradient has been applied to Wnt signaling in Zebrafish embryos [77]. This simulation takes into account key parameters in the formation of the gradient such as the length, angle distribution and growth dynamics of cytonemes. However, this simulation was made using Monte Carlo methods (computational algorithms based on repeated random sampling to obtain numerical results), but there are no mathematical or theoretical equations to support a cytoneme-based gradient formation model.

A purely theoretical model of cytoneme-mediated signaling suggested that this mechanism of delivery is more robust compared to passive diffusion mechanisms [128] (Figure 2D). However, several assumptions make its application very difficult in real biological systems. For instance, the model assumes that cytonemes are stable channels between cells and each source cell contacts via tubular connections with all target cells, each receiving a specific rate of cargo. According to this model, the rate of morphogen delivery across cytonemes plays a key role in gradient formation, even though these rates are not taken into account for their theoretical conclusion concerning robustness. These stable bridge-like structures are more biologically similar to nanotubes than to filopodia or cytonemes, due to their different cytoskeleton composition, their dynamics and their terminal ends.

Pattern formation based in cytoneme-mediated signaling has been modeled for the lateral inhibition of Notch signaling combining experimental and theoretical studies [81] (Figure 2E). To monitor Notch signaling, the authors use both live imaging and patterning of bristles in *Drosophila* adults, highlighting the role of cytonemes and their dynamics in bristles position. They also quantitatively reproduce by simulations of cytoneme and cell dynamics in a classical theoretical model of lateral inhibition [129]. Another theoretical study [130] describes Notch signaling using computer simulations of different mathematical weighting functions (in terms of homogenous or heterogeneous and symmetric or asymmetric signaling through nets of cytonemes). Despite the versatility of the model, there is not a direct link between mathematical parameters and biological variables. In addition, this work is restricted to static nets of cytonemes, while experimental studies show that cytonemes are dynamic structures [8,77,81].

Finally, there have been several works that combine biological and theoretical studies to analyze filopodia architecture and function [131,132,133], actin dynamics in filopodia [134,135] or active transport in filopodia [136], but none of them has been applied to filopodia-mediated signaling mechanisms, cytonemes or gradient formation.

In summary, several theoretical models in gradient formation have been proposed and improved over the years [118,119,137,138,139,140,141,142,143]. Large efforts are currently being made to develop new biophysical theories trying to model gradient formation at different levels taking into account the biological mechanism implicated in signaling processes [81,126,127,144,145] and the literature cited therein. Using our experimental system of cytoneme-mediated Hh gradient formation, we are generating data aiming to establish a more realistic biophysical model to describe gradient formation (Aguirre-Tamaral and Guerrero, CSIC-UAM, Madrid, Spain. Unpublished work, 2016).

## 8. Outlook on Cytonemes

Hh has to be transported from producing to receiving cells to activate its targets. Due to the particularity of Hh lipid moieties, several models have been proposed to explain Hh movement through the extracellular matrix. Although these models are not mutually exclusive, there is increasing evidence that the precise spatial control of Hh dispersion is most likely due to cytonemes-mediated transport, at least in *Drosophila* epithelia [8]. Cytonemes were proposed to ferry signaling proteins to deliver them to target cells through direct contacts [74] (reviewed in [6,146]). Experiments performed in the *Drosophila* wing air sac primordium have reported the existence of specific cytonemes dedicated to distinct signaling pathways [79].

Cytonemes mediating Hh transport have been visualized as dynamic structures and their extension correlates in space and time with gradient formation during development in *Drosophila* epithelia [8]. They act as the conduit of vesicles loaded with Hh to be delivered as exosomes at the contact sites [6]. Exchange of signals at specialized sites could mimic that of synaptic processes (González-Méndez, Seijo-Barandiarán and Guerrero, CSIC-UAM, Madrid, Spain. Unpublished work, 2016). It would be at these specialized sites where Hh and Ptc interact for reception. There are still uncertainties on where the contact actually occurs, if at the tip or along the whole length of both cytonemes. High-resolution confocal microscopy will bring information about the structure and composition of these contact sites.

Several issues regarding the mechanism of cytoneme growth and orientation have to be considered. Thus far, all cytoneme-mediated Hh transport has been observed oriented towards the receiving cells, which implies that cytonemes elongate towards their targets to make the correct connections. Conversely, the Hh receiving cells also extend cytonemes towards the morphogen source [8]. This mechanism is reminiscent of neuronal pathfinding processes, where guidance cues are needed to direct axons to their targets. Alternatively, cytonemes could extend in all directions and be stabilized when the appropriate connection is reached.

The dynamics of extension and retraction of cytonemes, both in *Drosophila* [8] and vertebrate tissues [10], indicates a prospective plasticity of these structures and gives an exciting dimension to the mechanism of morphogen gradient formation. Therefore, elements that could affect cytoneme formation and their dynamics may have an impact on signaling processes. For instance, regulators of actin dynamics and their coordination with the extracellular matrix components would be essential for gradient establishment. It is already known that the HSPGs of the extracellular matrix are important for the stabilization of cytonemes [8]. However, this requirement does not explain how cytonemes are committed to growing in a particular direction. Integrins and adhesion molecules might have a more active role in coordinating the cytoneme plasticity with the actin cytoskeleton dynamics.

Interestingly, the mechanism of signaling proteins transfer through sites of direct cell contact at a long distance is reminiscent of the contact-mediated signaling used by specialized cells like neurons. Axonal and cytoneme-mediated signaling share significant similarities, indicating that the mechanism might have a common origin. Alternatively, neurons could have evolved from a simpler cytoneme-like structure. Thus, previous knowledge on the synaptic process in neuronal cells could open up future investigations to further understand cytoneme function in cell–cell signaling.
